# Arginine deiminase pathway enzymes: evolutionary history in metamonads and other eukaryotes

**DOI:** 10.1186/s12862-016-0771-4

**Published:** 2016-10-06

**Authors:** Lukáš Novák, Zuzana Zubáčová, Anna Karnkowska, Martin Kolisko, Miluše Hroudová, Courtney W. Stairs, Alastair G. B. Simpson, Patrick J. Keeling, Andrew J. Roger, Ivan Čepička, Vladimír Hampl

**Affiliations:** 1 Department of Parasitology, Charles University, Faculty of Science, Prague, Czech Republic; 2 Department of Biochemistry and Molecular Biology, Dalhousie University, Halifax, Canada; 3 Institute of Molecular Genetics, Academy of Sciences of the Czech Republic, Prague, Czech Republic; 4 Department of Biology, Dalhousie University, Halifax, Canada; 5 Department of Botany, University of British Columbia, Vancouver, Canada; 6 Department of Zoology, Charles University, Faculty of Science, Prague, Czech Republic

**Keywords:** Arginine deiminase, Ornithine transcarbamylase, Carbamate kinase, Phylogeny, Metamonada, Preaxostyla, Protists

## Abstract

**Background:**

Multiple prokaryotic lineages use the arginine deiminase (ADI) pathway for anaerobic energy production by arginine degradation. The distribution of this pathway among eukaryotes has been thought to be very limited, with only two specialized groups living in low oxygen environments (Parabasalia and Diplomonadida) known to possess the complete set of all three enzymes. We have performed an extensive survey of available sequence data in order to map the distribution of these enzymes among eukaryotes and to reconstruct their phylogenies.

**Results:**

We have found genes for the complete pathway in almost all examined representatives of Metamonada, the anaerobic protist group that includes parabasalids and diplomonads. Phylogenetic analyses indicate the presence of the complete pathway in the last common ancestor of metamonads and heterologous transformation experiments suggest its cytosolic localization in the metamonad ancestor. Outside Metamonada, the complete pathway occurs rarely, nevertheless, it was found in representatives of most major eukaryotic clades.

**Conclusions:**

Phylogenetic relationships of complete pathways are consistent with the presence of the Archaea-derived ADI pathway in the last common ancestor of all eukaryotes, although other evolutionary scenarios remain possible. The presence of the incomplete set of enzymes is relatively common among eukaryotes and it may be related to the fact that these enzymes are involved in other cellular processes, such as the ornithine-urea cycle. Single protein phylogenies suggest that the evolutionary history of all three enzymes has been shaped by frequent gene losses and horizontal transfers, which may sometimes be connected with their diverse roles in cellular metabolism.

**Electronic supplementary material:**

The online version of this article (doi:10.1186/s12862-016-0771-4) contains supplementary material, which is available to authorized users.

## Background

The arginine deiminase pathway (ADI pathway, syn.: arginine dihydrolase pathway) catalyzes a conversion of arginine to ornithine, ammonium, and carbon dioxide, while generating ATP from ADP and phosphate. The enzymes involved in the three steps of the pathway are arginine deiminase (ADI, EC 3.5.3.6), ornithine transcarbamylase (OTC, EC 2.1.3.3), and carbamate kinase (CK, EC 2.7.2.2). The first reaction, catalyzed by ADI, is the deamination of arginine to yield citrulline and NH_4_^+^. OTC then catalyzes the conversion of citrulline and inorganic phosphate into carbamoyl-phosphate and ornithine. Finally, CK catalyzes the hydrolysis of carbamoyl phosphate to form CO_2_ and NH_4_^+^, while the phosphate group is used to regenerate ATP from ADP.

The ADI pathway is widely distributed among bacteria, where it is often a major means of energy production [1]. However, the ammonium produced by this pathway has also been implicated in protecting some bacteria from the harmful effects of acidic environments [2, 3]. The pathway has been also described in Archaea [4]. This pathway has only been characterized in a few species of anaerobic eukaryotes namely the parabasalids *Trichomonas vaginalis* [5] and *Tritrichomonas foetus* [6], and the diplomonads *Giardia intestinalis* [7], *Hexamita inflata* [8], and *Spironucleus salmonicida* [9]. All these species belong to Metamonada (Excavata), a clade of anaerobic protists with substantially modified mitochondria designated as hydrogenosomes or mitosomes. Metamonada consists of three lineages – Fornicata (e.g., *Giardia* and *Spironucleus*), Parabasalia (e.g., *Trichomonas* and *Tritrichomonas*), and finally Preaxostyla (*Trimastix*, *Paratrimastix* [10], and oxymonads) [11]. Currently, there is no information about the ADI pathway in Preaxostyla.

In *Trichomonas vaginalis* the ADI pathway generates up to 10 % of the energy produced by glucose fermentation [12]. OTC and CK were shown to be cytosolic, while ADI was described as membrane-associated in both *Trichomonas vaginalis* and *Tritrichomonas foetus* [6]. The ADI of *Trichomonas vaginalis* was later shown to be localized in hydrogenosomes and an *in situ* pH buffering function has been proposed [13]. The ADI pathway of *Giardia intestinalis* is completely cytosolic and produces up to 8 times more ATP than sugar metabolism [7]. Besides this energy-producing function, it has been proposed that the enzymes play an important role in the pathogenesis of *Giardia intestinalis* and *Trichomonas vaginalis*. The protists secrete ADI and OTC from their cells causing arginine depletion thus reducing the ability of the infected tissue to produce antimicrobial nitric oxide [14, 15]. Other known effects of these parasite's ADI pathway enzymes include growth arrest of intestinal epithelial cells [16], inhibition of T cell proliferation [15], and alteration of the phenotype and cytokine production of dendritic cells [17]. Another diplomonad with a characterized ADI pathway, the free-living *Hexamita inflata*, inhabits environments with varying levels of dissolved oxygen. It has been suggested that the ADI pathway may contribute to the metabolic flexibility of this organism, producing a significant amount of ATP under oxygen-limited conditions, while glycolysis is the main energy source under oxic or microoxic conditions, however the oxygen relationship might be incidental or secondary [8].

Of the three enzymes, only ADI itself is considered to be specific to the ADI pathway. CK has an additional role in purine and nitrogen metabolism and OTC may catalyze synthesis of citrulline as a nitrogen reservoir in plants [18] or be a part of ornithine-urea cycle in animals, diatoms and dinoflagellates [19, 20]. Therefore, the presence of ADI in organisms where no ADI pathway is known is intriguing and deserves further investigation. For example, within the chlorophytes the ADI gene was found in three species of *Chlorella* [21, 22] and *Chlamydomonas reinhardtii* [23] And ADI activity has been reported in multiple species of Chlorodendrophyceae, Trebouxiophyceae, Chlorophyceae, and Ulvophyceae [24], that is, in all classes of the “crown group” of Chlorophyta [25].

The first known sequence of a eukaryotic ADI, from *Giardia intestinalis*, showed no specific relationship to any bacterial or archaeal clade [26]. Later analyses included sequences from *Trichomonas vaginalis*, *Spironucleus vortens*, *Sp. barkhanus*, and *Sp. salmonicida* (Metamonada), *Euglena gracilis* and '*Seculamonas*' sp. (Discoba), *Chlamydomonas reinhardtii* and *Chlorella* sp. (core Chlorophyta [27]), and *Mastigamoeba balamuthi* and *Dictyostelium discoideum* (Amoebozoa). All the eukaryotic sequences formed a well-supported clade related to Archaea, consistent with a single origin of ADI in the eukaryotic domain [9, 13].

Due to its involvement in other pathways, it is not surprising that OTC is more widespread among eukaryotes compared to the other ADI pathway enzymes. The phylogenetic analysis of OTC by Zúñiga et al. [26] recovered two distinct eukaryotic clades branching in different positions among bacteria, one comprising sequences from Embryophyta and other composed of metazoan and fungal sequences. The only eukaryote outside these two clades was *Giardia intestinalis*, which was also the only one with a characterized ADI pathway. The sequence from *Giardia intestinalis* branched among bacterial sequences without close relationship to any other eukaryotic clade. Later analyses demonstrated that *Spironucleus salmonicida* and *Trichomonas vaginalis* OTC sequences formed a well-supported clade with *Giardia intestinalis* [9], suggesting the existence of a third independent group of eukaryotic OTCs present in Metamonada and potentially involved in the ADI pathway. The same analysis also showed two stramenopile sequences branching clearly inside the Metazoa-Fungi group.

Sequences of CK from *Giardia intestinalis*, *Hexamita* sp., and *Trichomonas vaginalis* formed a relatively well-supported clade not closely related to any bacterial or archaeal sequences [26]. The monophyly of eukaryotic CKs was later questioned after adding sequences from *Spironucleus salmonicida* and *Carpediemonas membranifera*, with the *Trichomonas vaginalis* sequence branching separately from other eukaryotes, although statistical support for this topology was very low [9].

In summary, the complete set of ADI pathway enzymes has been found in representatives of two out of three major lineages of Metamonada: Parabasalia and Fornicata. All the metamonad enzymes appear to be closely related to each other. This raises several questions about the evolutionary history of the pathway among eukaryotes. Is it present also in the third and least investigated lineage of metamonads, Preaxostyla? Was it present in the common ancestor of the Metamonada? Do representatives of other eukaryotic lineages possess the ADI pathway as well? If so, do all the eukaryotic enzymes involved in the ADI pathway originate from the same source or do they represent independent acquisitions?

Here, we take advantage of the recent progress in genome and transcriptome sequencing of less studied protists to perform an up-to-date survey and phylogenetic analysis of ADIs, OTCs, and CKs. This survey focuses on elucidating the evolutionary history of the arginine deiminase pathway in eukaryotes, with special emphasis on Metamonada. In addition to phylogenetic studies, we determine the subcellular localization of these enzymes in two members of Preaxostyla, *Paratrimastix pyriformis* and oxymonad *Monocercomonoides* sp. PA203.

## Results

### Distribution of ADI, OTC, and CK across eukaryotes

**Fig. 1:**
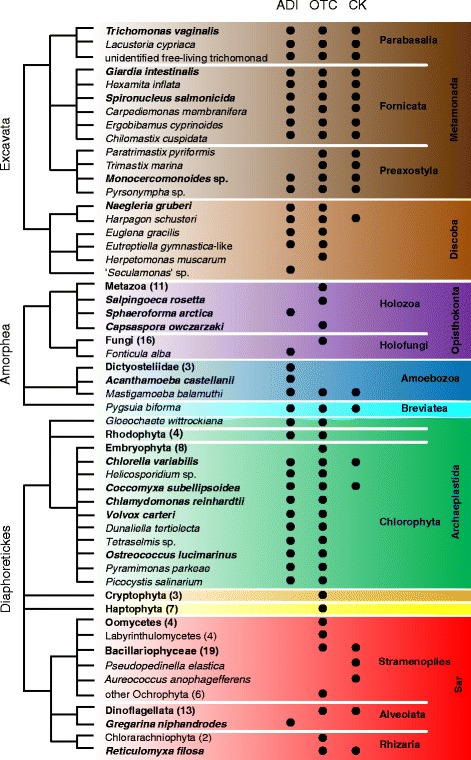
Distribution of enzymes of the arginine deiminase pathway across eukaryotic diversity. Arginine deiminase (ADI), ornithine transcarbamylase (OTC), carbamate kinase (CK). Taxon names in boldface indicate lineages containing at least one representative with a sequenced nuclear genome. Numbers in brackets indicate number of sequences from the given taxon included in our analyses. Colored rectangles indicate major eukaryotic groups as follows: *dark* *brown* – Metamonada; *light* *brown* – Discoba; *violet* – Opisthokonta; *blue* – Amoebozoa; *cyan* – Breviatea, *green* – Archaeplastida; *orange* – Cryptophyta; *yellow* – Haptophyta; *red* – SAR. Excavata, Amorphea, and Diaphoretickes are names of the 3 putative largest clades of eukaryotes as proposed in Adl et al., 2012; relationships between them are not resolved

Our survey revealed the presence of ADI, OTC, and CK in the three main eukaryotic clades defined by Adl et al., 2012 [28] (Fig. 1). The first and presumably the most specific enzyme of the pathway, i.e. without any role outside the ADI pathway reported so far, is ADI itself. This was found in 40 taxa, as shown on the schematic tree in Fig. 1, and these taxa represent most eukaryotic supergroups (highlighted by colored backgrounds). Of these, 16 species (most metamonads, *Harpagon*, *Mastigamoeba*, *Pygsuia*, *Chlorella*, and *Coccomyxa*) encoded all three enzymes, while the other species encoded only one or two enzymes. ADI was not detected in any representative of the clades Metazoa, Fungi, Embryophyta, Cryptophyta, and Haptophyta, nor in Sar [28], with the single questionable exception of *Gregarina niphandrodes* (see below). OTC was the most widespread enzyme, being found in 131 taxa including the major multicellular groups of Metazoa, Fungi, and Embryophyta. CK was detected in all the investigated metamonads, multiple Bacillariophyceae, Dinoflagellata and 8 other species. Please note that the given numbers do not represent the actual quantity of eukaryotic species with the particular gene since several groups, e.g. Metazoa, Bacillariophyceae, are represented by only a limited number of randomly selected sequences.

### Phylogenetic analyses

#### Arginine deiminase

**Fig. 2:**
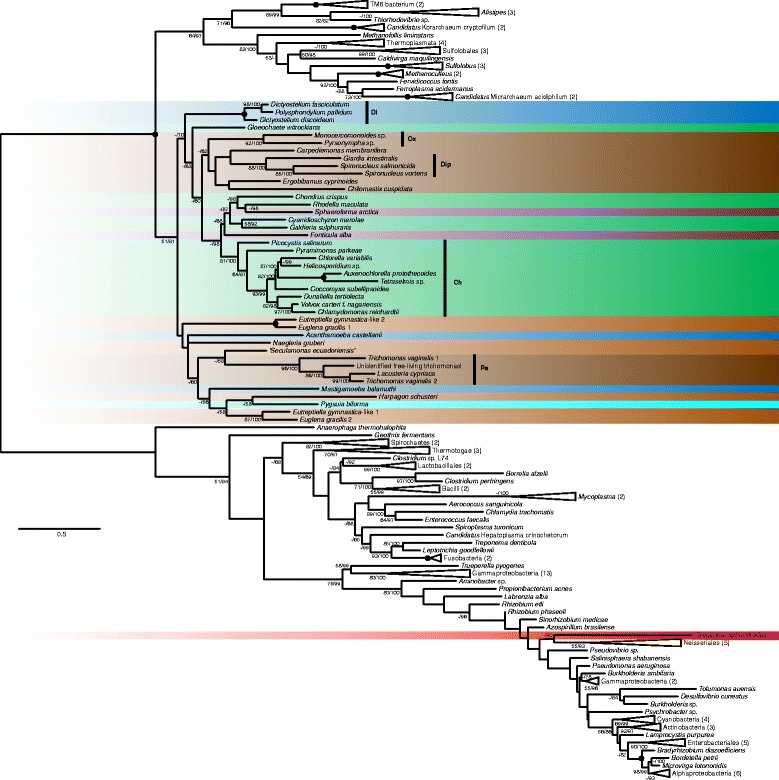
Phylogenetic tree of ADI sequences. The tree based on a 257 positions long protein alignment of 152 sequences was constructed in RAxML using the LG4X + Γ model of substitution. Eukaryotic taxa are highlighted in different colors according to the major group they belong to. The color code is the same as in Fig. 1. The values at nodes represent RAxML bootstrap support/IQ-TREE bootstrap support. Only values above 50 % are shown. *Black* circles indicate support of 100 %/100 %. Vertical *black* bars indicate well-supported eukaryotic clades: Pa – Parabasalia; Di – Dictyosteliida; Ch – Chlorophyta; Ox – Oxymonadida; Dip – Diplomonadida. Species with multiple sequences included: *Euglena gracilis* 1 – GI 109790819; *Euglena gracilis* 2 – GI 109784514; *Eutreptiella gymnastica*-like 1 – CAMPEP 0200414012; *Eutreptiella gymnastica*-like 2 – CAMPEP 0200422928; *Trichomonas vaginalis* 1 – TVAG 183850; *Trichomonas vaginalis* 2 – TVAG 344520. The tree is unrooted

Compared to the previous analyses we present a more robust analysis including 40 eukaryotic species (Fig. 1). The phylogenetic tree (Fig. 2) shows two clearly separated (RAxML bootstrap support/IQ-TREE bootstrap support: 100 %/100 %) clans of ADIs, one comprising all bacteria and one isolated eukaryote, *Gregarina niphandrodes*, in a highly nested but poorly resolved position, and the second composed of clans of Archaea plus a few Bacteria (64 %/97 %) and Eukaryota (51 %/81 %). The topology within the eukaryotic branch is poorly resolved overall, however, a few clades of lower-than-supergroup rank were recovered with strong support (i.e. bootstrap support > 80 %). These are Parabasalia, Diplomonadida, Oxymonadida, Chlorophyta, and Dictyosteliida.

### Ornithine transcarbamylase

Our analysis included sequences from selected representatives of Metazoa, Fungi, and Embryophyta and 110 sequences from 103 other eukaryotes (Fig. 1). Several bacterial sequences of aspartate transcarbamylase, a protein closely related to OTC, were included to provide an outgroup for rooting the tree. Our analysis of OTC phylogeny (Additional file 1) supports the existence of three large groups and two separately-branching eukaryotic OTCs.

The first large clade is strongly supported (100 %/100 %) and contains Metazoa, Fungi, Oomycota, Bacillariophyceae (i.e. diatoms), a few lineages of other Stramenopiles (*Nannochloropsis*, *Vaucheria*, *Ectocarpus*, *Heterosigma* and *Ochromonas*), the holozoan *Capsaspora*, and one single excavate, the trypanosomatid *Herpetomonas muscarum*, which branches among Fungi.

The second eukaryotic group, already indicated in the analysis by Andersson et al. [9], is well supported (83 %/100 %) and includes all Metamonada, *Harpagon shusteri*, *Naegleria gruberi, Reticulomyxa filosa*, *Pygsuia biforma*, *Mastigamoeba balamuthi*, and many representatives of autotrophic groups, namely dinoflagellates, cryptophytes, euglenophytes, chlorarachniophytes, and stramenopiles like *Aureococcus*, *Aureoumbra*, *Pelagococcus*, *Pelagomonas*, and *Pseudopedinella*. The monophyly of Parabasalia is well supported. A sequence from a recently described archaeon *Lokiarchaeum* sp. is also included in this group, however at an unsupported position.

The third group is composed of euglenophytes, green algae with green plants, red algae, and haptophytes, with haptophytes branching inside red algae. A clade of mostly Desulfobacteraceae bacterial sequences branches inside this group of eukaryotic sequences.

The only two eukaryotes outside these three large clades are the choanoflagellate *Salpingoeca rosetta* (sequence obtained from the genome), which branches as sister to Microgenomates bacterium (78 %/100 %), and the rhizarian *Paulinella chromatophora* (red star in Additional file 1) inside Cyanobacteria with good statistical support (100 %/100 %). Since the *Paulinella* sequence originates from the genome of the chromatophore, not the *Paulinella* nucleus, it actually represents a cyanobacterial OTC.

### Carbamate kinase

We have included 47 sequences from 44 eukaryotic species in our analysis (Fig. 1). Our tree (Additional file 2) shows eukaryotes falling into several separate clusters. One of two substantial groups is an unsupported clan of Fornicata, Parabasalia, *Harpagon schusteri*, *Pygsuia biforma*, and *Mastigamoeba balamuthi*. A well-supported Preaxostyla clade (96 %/100 %) branches at a different place among bacteria, as a sister group to Hadesarchaea archaeon and Anaerolineae bacterium (96 %/100 %). The second large eukaryotic clan (100 %/100 %) is composed of all the dinoflagellate sequences, as well as sequences from diatoms, *Pedinella* and *Aureococcus*. Dinoflagellata form a well-supported group within this clan. Three sequences from diatoms do not branch together with other ochrophytes (the photosynthetic Stramenopiles), and instead form a separate well-supported clan (100 %/100 %) among bacteria. This may represent a second form of the enzyme, since *Thalassiosira pseudonana* appears in both diatom groups. The only two known CKs from green plants (*Chlorella* and *Coccomyxa*) branch together (97 %/99 %) but separated from other eukaryotes. The *Reticulomyxa* CK sequence is also isolated from the rest of eukaryotes.

### Concatenation

We also performed a phylogenetic analysis of a concatenation of all three enzymes. In the first step, we have prepared an alignment supermatrix in which we have included all eukaryotes and representatives of prokaryotes that contain a complete set of the three enzymes, and may use the ADI pathway. In order to detect potential incongruities between gene partitions caused by lateral gene transfer we have performed a phylogenetic analyses of the individual gene partitions from this supermatrix. Based on these gene trees (Additional file 3) we removed taxon-gene sequences that branched with bootstrap support higher than 50 % within a clan of sequences outside its own domain (e. g. eukaryotic sequence outside Eukaryota) from the concatenated alignment – namely CKs from *Monocercomonoides* sp., *Pyrsonympha* sp., *Chlorella variabilis*, and *Coccomyxa subelipsoidea*. We also removed OTCs from *Chlorella variabilis*, and *Coccomyxa subelipsoidea* because in the large single gene tree (Additional file 1) they branch within a clade which is sister to Chlorobi Bacteria with 100 % IQ-TREE bootstrap support.

**Fig. 3:**
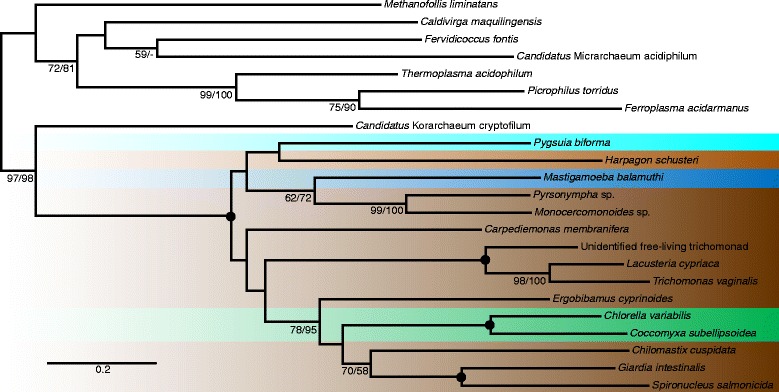
Phylogenetic tree of concatenated ADI, OTC, and CK sequences with Bacteria removed. The tree based on a 750 positions long protein alignment of 23 sequences was constructed in RAxML using LG4X + Γ model. Eukaryotic taxa are highlighted in different colors according to the major group they belong to. The color code is the same as in Fig. 1. The values at nodes represent RAxML bootstrap support/IQ-TREE bootstrap support. Only values above 50 % are shown. *Black* circles indicate support 100 %/100 %. The tree is unrooted

The analysis performed on the alignment after removal of these sequences (Additional file 4) revealed a strong bipartition (100 %/100 %) grouping Eukaryota and Archaea to the exclusion of Bacteria and within this part of the tree the eukaryotes formed a well-supported (100 %/100 %) clan sister to the archaeon *Candidatus* Korarchaeum cryptofilum. In order to recover the relationships within the Eukaryota–Archaea group without the disturbing long branch of Bacteria we repeated the analysis without the bacterial sequences (Fig. 3). In this unrooted tree Eukaryota are grouped with *Candidatus* Korarchaeum cryptofilum to the exclusion of the rest of Archaea with high support (97 %/98 %). We also performed a Eukaryota-only analysis of the concatenated dataset for the purpose of hypotheses testing (Additional file 5).

### Hypotheses testing

We used approximately unbiased (AU) and expected likelihood weight (ELW) tests to assess whether the inferred phylogenies are in a significant conflict with the monophyly of eukaryotes, metamonads and with the expected eukaryotic species tree. The results are summarized in Table 1. The AU tests rejected monophyly of metamonads in the OTC and CK trees and monophyly of expected eukaryotic phylogeny in concatenation. The ELW tests rejected the monophyly of metamonads in the OTC tree, the monophyly of both clades in the CK and monophyly of metamonads and the expected eukaryotic phylogeny in the concatenation tree.

**Table 1:** Results of approximately unbiased (AU) and expected likelihood weights (ELW) tests

Data set – hypothesis	AU test	ELW test
ADI – Metamonada monophyly.	0.64	0.10
OTC – Eukaryota monophyly	0.64	0.5
OTC – Metamonada monophyly	0	0
CK – Eukaryota monophyly	0.12	0
CK – Metamonada monophyly	0	0
Euk. Conc. – Metamonada monophyly	0.17	0
Euk. Conc. – expected euk. phylogeny	0	0

The tests were performed for 4 sets of taxa – ADI: ADI dataset (as in Fig. 2), OTC: OTC dataset (as in Additional file 1), CK: CK dataset (as in Additional file 2), Euk. Conc.: concatenation dataset without prokaryotic sequences (as in Additional file 5)

### Localization of ADI pathway enzymes in Preaxostyla

Another aim of this work was to infer the subcellular localization of ADI pathway enzymes in members of the poorly studied Preaxostyla clade. Genomic and transcriptomic projects have revealed the presence of all three enzymes in *Monocercomonoides* sp. and *Pyrsonympha* sp., while only OTC and CK enzymes were detected in *Trimastix marina* and *Paratrimastix pyriformis*. We have chosen *Monocercomonoides* sp. PA203 and *Paratrimastix pyriformis* for further study.

We investigated the presence of mitochondrion-targeting signals in the enzymes of interest (Additional file 6) using the signal prediction software TargetP 1.1 [29] and Mitoprot II v1.101 [30]. TargetP did not predict any targeting signals. Mitoprot II predicted a single mitochondrion-targeting signal, for the OTC sequence of *Paratrimastix pyriformis*.

**Fig. 4:**
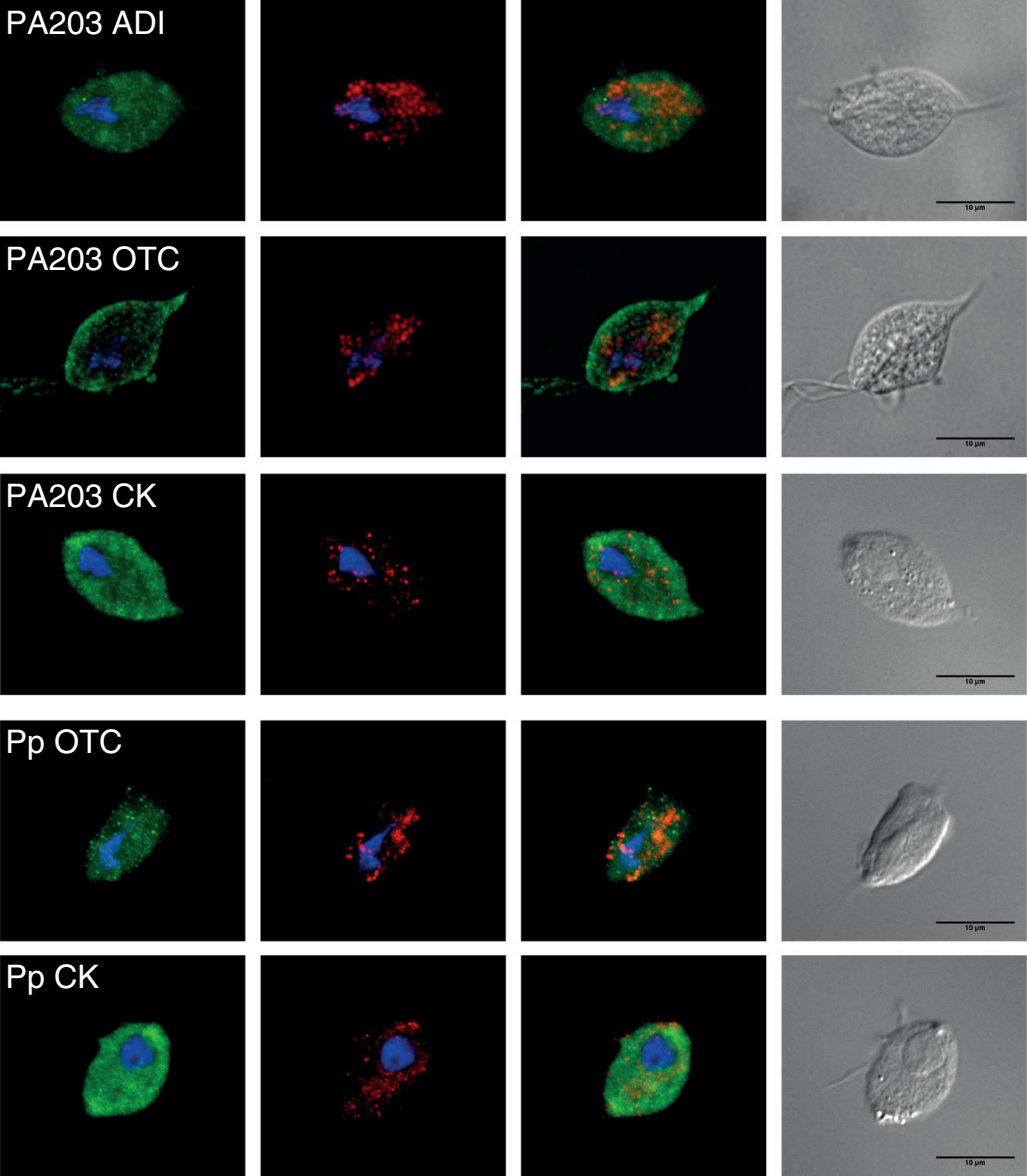
Localisation of *Monocercomonoides* and *Paratrimastix* enzymes in *Trichomonas vaginalis* cells. Immunofluorescence micrographs of *Trichomonas vaginalis*, in which the HA-tagged versions of enzymes were expressed. *Green* signal from anti-HA antibody does not localize to hydrogenosomes of *Trichomonas vaginalis*, which are marked by *red* anti-malic enzyme antibody. *Blue* signal indicates DAPI-stained nuclei. Abbreviations: PA203 – *Monocercomonoides* sp. PA203; Pp – *Paratrimastix pyriformis*; ADI – arginine deiminase; OTC – ornithine transcarbamylase; CK – carbamate kinase

To validate the results of mitochondrion-targeting signal prediction we used the *Trichomonas vaginalis* T1 heterologous expression system, with the assumption that an undetected mitochondrion-targeting signal may nonetheless be recognized by the *Trichomonas* hydrogenosomal import machinery. We transfected *Trichomonas vaginalis* cells with plasmids containing HA-tagged OTC and CK from *Paratrimastix pyriformis* and ADI, OTC, and CK from *Monocercomonoides* sp. In all cases fluorescence microscopy showed that the heterologously expressed proteins do not co-localize with the signal from the hydrogenosomal marker protein (malic enzyme), but instead formed a diffuse pattern all over the cell (Fig. 4). This demonstrates that the inserted proteins are not recognized as hydrogenosomal-import targets in *Trichomonas vaginalis*. The results of these experiments are consistent with the fact that most ADI pathway enzymes in eukaryotes are localized in the cytosol.

## Discussion

### ADI pathway enzymes are widespread in eukaryotes

Before this study, only two closely related lineages of eukaryotes had been conclusively shown to possess a complete ADI pathway. These were Parabasalia and Diplomonadida, both being members of Metamonada, a subgroup of Excavata. Our survey has shown the presence of all three enzymes in ten other eukaryotic species. Among these are other members of Metamonada – including free-living members of Fornicata related to the predominantly parasitic diplomonads (*Ergobibamus cyprinoides*, *Chilomastix cuspidata, Carpediemonas membranifera*), and members of the third metamonad lineage, Preaxostyla (*Monocercomonoides* sp. and *Pyrsonympha* sp.). The ADI pathway was also identified in non-metamonads including the heterolobosean *Harpagon schusteri*, the amoebozoan *Mastigamoeba balamuthi*, the breviate *Pygsuia biforma*, and the green algae *Chlorella variabilis* and *Coccomyxa subelipsoidea*. Further functional studies are needed to determine whether these enzymes function within an ADI pathway in these species. It is possible that the possession of the complete pathway is connected with their anaerobic lifestyle since most of these organisms are anaerobes, microaerophiles or aerobes able to live for long periods under anaerobic conditions [31–37].

Many investigated eukaryotes possessed incomplete sets of ADI pathway enzymes. The presence of OTC or CK on their own is not surprising, as they are known to be involved in other biochemical processes including the ornithine-urea cycle or purine biosynthesis. The presence of ADI on its own was unexpected, yet we identified ADI in a broad spectrum of eukaryotic lineages without complete pathway. The apparent absence of OTC or CK may be due to the incompleteness of transcriptome or genome data, nevertheless, our observation suggests that ADI may also function outside the context of the ADI pathway in some eukaryotes.

### Phylogenetic histories of enzymes

None of the enzyme phylogenies is completely consistent with the expected species relationships. In single gene trees, eukaryotes are always dispersed in multiple clades, suggesting complicated evolutionary histories. The backbone topologies were generally weakly supported, and many of these incongruences are probably the result of low phylogenetic signal. Nevertheless, some conflicts with species phylogeny are better supported and some were confirmed by phylogenetic tests. These can potentially be attributed to lateral gene transfers (LGTs, also known as horizontal gene transfers – HGTs) or endosymbiotic gene transfers (EGTs). The sister relationship of Preaxostyla and *Spirochaeta* in the CK tree, and the position of *Gregarina* within Bacteria in the ADI tree are two such examples of potential LGT, albeit the latter may also represent a contamination. Since haptophytes are known to harbor secondary plastids of potentially red algal origin [38], the position of haptophytes within a red algal clade in the OTC analysis, might represent a potential EGT. Conversely, many moderately and robustly supported eukaryotic clades are taxonomically reasonable, indicating the important role of vertical inheritance.

The taxon sampling in the concatenation analyses was lower, because the analyses included only those taxa that may utilize the arginine deiminase pathway and not those that use individual enzymes for other purposes. The resolution of the concatenation tree was higher than the individual gene trees and strongly supported the monophyly of eukaryotes (99 %/100 %) and their close relationship to Archaea. The clade of eukaryotes branched with Archaea (100 %/100 %) as a sister to *Candidatus* Korarcheum cryptophylum. Increased support of these nodes should partly be ascribed to the lower number of taxa but it also suggests that the phylogenetic signal regarding these deep nodes for this narrow set of taxa and after exclusion of obvious cases of LGT is largely congruent.

### ADI pathway is ancestral in Metamonada

The presence of the complete ADI pathway is widespread in metamonads, protists that specifically inhabit low-oxygen environments. In most of the phylogenies, metamonad taxa branch close to each other, but they never form an exclusive clade. In CK trees there is a well-supported relationship between Preaxostyla (*Paratrimastix*, *Monocercomonoides,* and *Pyrsonympha*) sequences and a prokaryotic clan of Hadesarchaea archaeon and Anaerolineae bacterium. Metamonada do not appear monophyletic even in the concatenation trees and their monophyly was rejected by ELW test. Taking together all this information we propose that the complete arginine deiminase pathway was present already in the common ancestor of Metamonada and was vertically inherited by the extant metamonad lineages, with a few exceptions. The exceptions are the putative losses of ADI in *Trimastix marina* and *Paratrimastix pyriformis* and the putative replacement by a bacterial CK in the Preaxostyla. It is also possible that some enzymes or the whole pathway were laterally transferred from metamonads to other eukaryotes, which would disrupt the monophyly of Metamonada on trees.

Our localization experiments suggest that all enzymes in both *Paratrimastix pyriformis* and *Monocercomonoides* sp. are localized in the cytosol, like the enzymes in *Giardia intestinalis* but unlike the ADI in *Trichomonas vaginalis*. We therefore conclude that cytosolic localization of the pathway is an ancestral trait of all metamonads. It should be noted, however, that the cytosolic localization of ADI in *Monocercomonoides* sp. may not be informative, since this protist does not contain mitochondrion [39]. *Paratrimastix pyriformis* harbors an organelle similar to the hydrogenosome, but no ADI-coding gene has been found in its transcriptome to test its localization.

### ADI pathways in other eukaryotes

Besides Metamonada, five other species contain a complete ADI pathway and all branch within the eukaryotic clade on the concatenation tree (Fig. 3). These represent four different supergroups of eukaryotes; only *Harpagon* is from the same supergroup as Metamonada (supergroup Excavata), and even then it belongs to a different sub-branch (Discoba). Moreover, Excavata are likely not monophyletic at all [40] and the root of eukaryotes may be situated inside the group. *Harpagon*, *Mastigamoeba*, and *Pygsuia* ADI pathway enzymes branch close to Metamonada in all three gene trees, and so it is very probable that the pathway in these three species was derived from the same source as the pathway in Metamonada.

The situation in *Chlorella variabilis* and *Coccomyxa subelipsoidea* is less clear. These organisms are the only green algae known to contain all three ADI pathway enzymes. Their ADIs and OTCs branch together with other green algae and plants in the individual gene trees (while no other Plantae have CK), supporting the presence of the complete set in the last common ancestor of Chlorophyta. However, Chlorophyta did not branch in a common eukaryotic clade with Metamonada, *Harpagon*, *Mastigamoeba* and *Pygsuia* in OTC and CK phylogenies, suggesting that these two enzymes in Chlorophyta may have independent origins. In the concatenation tree, *Chlorella variabilis* and *Coccomyxa subelipsoidea* represented only by ADI sequences branch together with other eukaryotes. Presence of the ADI pathway in the last common ancestor of Chlorophyta would be consistent with the ADI enzymatic activity previously reported from members of Chlorodendrophyceae, Trebouxiophyceae, Chlorophyceae, and Ulvophyceae [24]. However, the function of the other enzymes in a typical ADI pathway is questionable, since the OTCs from *Chlorella autotrophica*, *Chlorella saccharophila* (Trebouxiophyceae), and *Dunaliella tertiolecta* (Chlorophyceae) were found to have no measurable activity in the direction of the ADI pathway, i.e. conversion of citrulline to ornithine [24]. It is therefore possible that the two Chlorophyta species with all three enzymes nonetheless do not use the ADI pathway.

### Origin of the eukaryotic ADI pathway

The simplest explanation of the fact that the complete sets of ADI pathway enzymes from several eukaryotic lineages are related is that they are inherited from their common ancestors. The taxonomic composition of the eukaryotic clade in the concatenation tree is so broad that their common ancestor must have been either the last eukaryotic common ancestor (LECA) or its close descendant. This assumption is reasonable even if we would not consider *Chlorella* and *Coccomyxa*. An alternative explanation for the close relationships of ADI pathways would be that the genes were acquired more recently by one eukaryotic lineage (perhaps Metamonada, where it is most common), and then spread from this lineage into others via eukaryote-to-eukaryote lateral gene transfers.

Based on our data we are unable to decide which alternative is more likely. Vertical inheritance of the ADI pathway from LECA would be consistent with the sisterhood of the eukaryotic clade and the archaeon *Candidatus* Korarchaeum cryptofilum in the concatenation tree (Fig. 3, Additional file 4), since recent studies indicate Korarchaeota are indeed closely related to the eukaryotes [41]. Moreover, OTC sequences from *Lokiarchaeum* sp.*,* which is the closest known relative to eukaryotes [42], are related to the Metamonada-containing eukaryotic clade. CK sequence of this archaeon branches outside eukaryotes sister to a proteobacterium *Desulphobacula toluolica* (97 %/100 %), but nodes separating these two from Metamonada did not receive strong support. ADI sequence from *Lokiarcheum* is not available, and so this organism was not included in the concatenation analysis. We must also take into account that the position of the root of the eukaryotic tree is still an unresolved question [40, 43, 44]. If the root falls outside Amorphea + Discoba (position of Metamonada relative to the root is not known), then our results do not necessarily uncover the condition of LECA.

The later acquisition of the pathway by Metamonada and its spread to unrelated eukaryotes by eukaryote-to-eukaryote transfer is supported by the fact that the concatenation trees are incongruent with the expected relationships of taxa.

## Conclusions

Our broad survey of the arginine deiminase pathway enzymes has shown that they are present in representatives of all major lineages of eukaryotes. Sixteen protists (most metamonads, *Harpagon*, *Mastigamoeba*, *Pygsuia*, *Chlorella*, and *Coccomyxa*) contain the complete set of the three enzymes, while other organisms contain incomplete sets. The enzyme ADI is present in several species without the complete arginine deiminase pathway, suggesting its involvement in other cellular processes. The topology of individual gene trees is generally not very well supported and particularly in OTC and CK trees the eukaryotic enzymes form multiple clearly unrelated clades consisting of mixtures of eukaryotic supergroups. This indicates that multiple prokaryote-to-eukaryote and eukaryote-to-eukaryote LGT events took place in the history of these enzymes. It is possible that in some groups the enzyme acquisition was connected with its involvement in novel biochemical processes like the ornithine-urea cycle.

Based on the presence of the complete pathway in most metamonads and based on the phylogenetic affinity of metamonad enzymes, we conclude that the ancestor of metamonads already possessed this pathway. The concatenation analyses suggest that eukaryotes with the complete ADI pathway, including Metamonads, *Harpagon*, *Mastigamoeba* and *Pygsuia* (and possibly *Chlorella* and *Coccomyxa*), may have acquired the genes from a single, archaeon-related source. One intriguing possibility is that the acquisition of the pathway may date back as deep as to LECA, but other scenarios involving LGT events are also plausible. To resolve the last issue, it will be necessary to obtain data from a more diverse set of prokaryotes and eukaryotes, especially those branching close to the root of the eukaryotic tree and close to the root of major eukaryotic lineages.

## Methods

### Obtaining sequences

The majority of eukaryotic sequences included in the survey were obtained from the NCBI database (Release 68), JGI database [45], or Marine Microbial Eukaryote Transcriptome Sequencing Project [46]. Initial searches were performed using BLASTp and tBLASTn algorithms [47] with *Giardia intestinalis, Trichomonas vaginalis*, and several bacterial sequences as queries. The searches of public databases were then repeated several times while restricted to a particular major eukaryotic lineage (e.g. Cryptophyta, Alveolata) and with a phylogenetically closest available sequence as a query. All the eukaryotic sequences with E-value lower than 10^−3^ were downloaded and used in subsequent analyses.

The prokaryotic sequences included in the survey were retrieved from the NCBI database using the same query as in the search for eukaryotic sequences. The search was also repeated several times with varying taxonomic restrictions to ensure that all the bacterial and archaeal phyla containing the particular enzyme are represented in the analysis. We used all archaeal sequences with E-value lower than 10^−3^ and a limited number of bacterial sequences with E-value lower than 10^−3^ and annotated as the protein of interest. It is important to note that the set of bacterial sequences used in our analyses is not exhaustive and therefore we do not infer any evolutionary hypotheses about Bacteria in this study.

In order to mitigate the risk of missing prokaryotic data influencing the relationships between eukaryotic groups we enriched our datasets with the closest prokaryotic homologs to each of the eukaryotic sequences by searching the NCBI database using BLASTp with each eukaryotic sequence as a query and downloaded the prokaryotic sequence with the lowest e-value from each search.

We investigated whether those eukaryotic sequences which were not branching within eukaryotic clades represent *bona fide* eukaryotic sequences or contamination of the data sets. Nucleotide sequences obtained from transcriptomic data were checked for similarity with sequences deposited in NCBI and those that were identical or very similar to bacterial genomes (bit score higher than 200 and similarity along the entire length of the sequence) were excluded. In the case of sequences obtained from genomes, the entire gene content of the contiguous sequence scaffold was used as a query for BLAST search of the NCBI database in order to identify any known sequences with high sequence similarity along the entire length of the sequence, indicating possible contamination. Furthermore, candidate sequences and neighboring genes were investigated for the presence of introns and the origin and annotation of surrounding genes. These steps should identify some sequences originating from contamination, however, others could still remain due to the lack of data from the source of the contamination or incorrect assembly of genomic data resulting in chimerical sequences.

The sequences downloaded from public databases were combined with sequences extracted from genomic and transcriptomic projects performed in the laboratories of co-authors. Brief information on the generation of these data sets is given below. Details of the *Monocercomonoides* sp. PA203 genome and transcriptome project are given in Karnkowska et al. [39]. Details of the *Paratrimastix pyriformis* transcriptome project are given in Zubáčová et al. [48]. Partial cDNA sequences corresponding to *Paratrimastix pyriformis* OTC and CK obtained in the transcriptome project were completed at their 5` ends by RACE using FirstChoice RLM-RACE kit (Life Technologies, AM1700). Amplifications by PCR were carried out using Takara Hot-Start ExTaq DNA Polymerase (Takara, RR006A) in 50 μl reactions. Outer 5` RLM-RACE PCR was done using the 5` RACE outer primer supplied in the kit and the following 5` RACE gene-specific outer primers: TpOTCout: CCAGCAGGAAGAGAAGGAGG and TpCKout: GCTTGCCGTAGTTGATGATG. Inner 5` RLM-RACE PCR was done using the 5` RACE inner primer supplied in the kit and the following 5` RACE gene-specific inner primers: TpOTCinn: AAGAGCTCGTGATCTGGAAG and TpCKinn: GCCAGAGGCGATGACAATGA (here and elsewhere, all primers reported in the 5’ to 3’ direction). The following touchdown program was used for each of the two PCRs: 95 °C (5 min), 15 cycles of 95 °C (1 min), 60 °C to 45 °C (35 s.) and 72 °C (2 min), 20 cycles of 95 °C (1 min), 45 °C (35 s.) and 72 °C (2 min), then a final polymerization step at 72 °C for 6 min. PCR products were cloned into pGEM-T Easy plasmid vector (Promega, A1360) and sequenced.

Sequences from *Lacusteria cypriaca* (strain LAI), an unidentified free-living trichomonad (strain LAGOS2D) and *Pyrsonympha* sp. were mined from RNA-seq data sets generated using the llumina MiSeq sequencing platform. Sequences from *Trimastix marina*, *Carpediemonas membranifera*, *Chilomastix cuspidata*, *Mastigamoeba balamuthi*, and *Pygsuia biforma* were mined from RNA-seq data sets generated using the Illumina HiSeq sequencing platform. Sequences from *Harpagon schusteri* were mined from RNA-seq data obtained using the 454 sequencing platform and sequences of *Ergobibamus cyprinoides* were mined from data sets generated by combination of Sanger and 454 sequencing platforms. The assembled sequences were submitted to GenBank under accession numbers KT883858-KT883885.

### Phylogenetic analyses

Inferred amino acid sequences were aligned using MAFFT version 7 [49] and the resulting alignments were manually trimmed. Highly variable misaligned sections of several eukaryotic sequences, possibly results of sequencing errors, were removed from the alignment manually. The concatenated alignment was constructed from the single gene alignments using SequenceMatrix [50]. The final alignments can be downloaded from our web page: http://protistologie.cz/hampllab/NovakData.zip [51]. Phylogenetic inference was performed using substitution models suggested by ProtTest 2.4 server [52] – LG4X + Γ model. Maximum Likelihood trees were inferred using RAxML-HPC2 version 8 available on CIPRES [53], with 10 starting trees and also using IQ-TREE v1.4.2 [54] under the LG + C20 + F + G4 model for the single gene trees and LG + C40 + F + G4 model for concatenated datasets. The model that best fits the data was determined by IQ-TREE according to the Bayesian information criterion (BIC). The LG matrix was combined to an amino acid class frequency mixture model with 20 (for single gene trees) and 40 (for concatenated datasets) frequency component profiles. Statistical support for branches was assessed by multiparametric bootstrapping (1000 replicates) in RAxML and by the ultrafast bootstrap approximation (UFboot) with 1000 replicates in IQ-TREE.

### Topology tests

Phylogenetic hypotheses were tested by an approximately unbiased (AU) test [55] and expected likelihood weights (ELW) method [56] implemented in IQ-TREE 1.4.2 [54]. For all datasets we tested whether their ML phylogeny is in significant conflict with the monophyly of Metamonada. In the case of OTC and CK, we also tested whether the phylogeny is in significant conflict with the monophyly of eukaryotes. In the case of the concatenated dataset, we tested whether their ML phylogeny is in significant conflict with the monophyly of Metamonada as well as whether the relationships within the eukaryotic clade (Additional file 5) significantly conflicts with a user-defined expected species tree (Additional file 7). For the latter two tests we used a concatenated dataset without prokaryotic sequences to eliminate the influence of relationships among prokaryotes and position of the eukaryotic root. The ADI sequence attributed to *Gregarina niphandrodes* was excluded from all tests including ADI data, as this clearly represents contamination or very recent LGT.

To perform AU and ELW tests, a set of 1003 topologies was created, containing the unconstrained ML topology inferred by RAxML, 1000 topologies inferred by RAxML during bootstrapping, and the best trees inferred by RAxML under the selected constraints (eukaryotic monophyly, Metamonada monophyly or eukaryotic phylogeny). Site likelihoods for topologies were calculated by IQ-TREE using the LG + C20 + F + G4 model. The sets of site likelihoods were then compared using the AU test in IQ-TREE, with 10 000 replicates.

### Cloning of ADI pathway genes

ADI pathway genes were amplified from *Paratrimastix* and *Monocercomonoides* cDNAs by PCR. *Paratrimastix pyriformis* and *Monocercomonoides* sp. PA203 cDNAs were prepared from 100 mL of bacterized culture and 1000 mL of culture filtered according to Hampl et al. [57], respectively. Isolations of total RNA were performed using TRI Reagent RNA Isolation Reagent (Sigma, T9424). Extractions of eukaryotic mRNA from total RNA were done using a Dynabeads mRNA Purification Kit (Life Technologies, 61006). The SMARTer PCR cDNA Synthesis Kit (Clontech, 634925) was used for cDNA synthesis following by cDNA amplification with an Advantage 2 PCR Kit (Clontech, 639207) using 21 cycles (*Paratrimastix*) and 19 cycles (*Monocercomonoides*) of amplification.

The following primers were used for amplifications of full-length cDNAs of the ADI pathway genes of *Paratrimastix* and *Monocercomonoides* (restriction sites NdeI, VspI, and BamHI are in bold): Tp OTC-F (TAA**CATATG**CCTCGCCACCTTACCAAGAT), Tp OTC-R (TAA**GGATCC**GTCAAGGAGGGGCTGGCCCA), Tp CK-F (TAA**CATATG**CGTATCCTCATCGCTCTCG), Tp CK-R (TAA**GGATCC**GGCGACAATGTGGGTACCAG), PA203 ADI-F (TAA**CATATG**ATGCAAGATATTCACGTTCC), PA203 ADI-R (TAA**GGATCC**CTGATTTCCCAGAGATGCTA), PA203 OTC-F (ATC**ATTAAT**ATGTCCGCTCCCGTTAGACA), PA203 OTC-R (TAA**GGATCC**CTCAATGGTCATTTTCTTGT), PA203 CK-F (CACTTCACATTA**CATATG**GTGAGAATTTTAATTGCTC), PA203 CK-R (CGTATGGGTA**GGATCC**TGGAACAATGTGAGTTCCTT). For transfection of *Trichomonas vaginalis,* the genes were cloned into TagVag2 plasmid vector [58] using restriction digestion and ligation, or directly using the In-fusion HD Cloning Kit (Clontech, 639648) in the case of *Monocercomonoides* CK. Lab-grown chemically competent *Escherichia coli* XL1 cells were used for transformations with ligation mixtures, whereas Stellar competent cells (Clontech, 636763) were used for transformation with the in-fusion reactions. Bacterial clones were checked by colony PCR for the presence of the plasmids followed by sequencing of isolated plasmids.

### Selectable transfection of *Trichomonas vaginalis*

Despite extensive efforts, we did not achieve either stable or transient transfection of *Paratrimastix pyriformis* and *Monocercomonoides* sp. PA203 with plasmid vectors specifically prepared for those two organisms (data not shown). Therefore, the *Trichomonas vaginalis* heterologous expression system was used to infer the subcellular localizations of the *Paratrimastix* and *Monocercomonoides* enzymes. Versions of ADI pathway genes with a C-terminal 2xHA-tag were electroporated into *Trichomonas* cells according to the protocol described by Sutak et al. [59]. Briefly, 250 mL of *Trichomonas vaginalis* T1 culture (strain kindly provided by Michaela Marcinčiková, Dept. of Parasitology, Charles University) was used for two electroporations performed for each of the genes. Cells were electroporated with 30 μg of TagVag2 plasmid isolated using the Wizard Plus Midipreps DNA Purification System (Promega, A7640). The exponential protocol (350 V, 975 μF, ∞ Ω, 4 mm cuvette) of the GenePulser Xcell Electroporation System (Biorad, 165–2660) was used for each transfection. Trichomonads were selected with 200 μg/ml of G418 (ZellBio, G-418-5) for at least five passages. Expression of the proteins was analyzed by Western blotting of cell homogenates (data not shown) and immunofluorescence microscopy with antibody.

### Immunofluorescence microscopy

ADI pathway proteins of *Paratrimastix* and *Monocercomonoides* were identified in *Trichomonas* cells using an anti-HA rat monoclonal antibody (Roche, 11867423001). An antibody raised against malic enzyme, a hydrogenosomal marker in *Trichomonas vaginalis* [60], was used for double-labeling (antibody kindly provided by prof. Jan Tachezy, Dept. of Parasitology, Charles University). Alexa Fluor-488 goat anti-rat (green) and Alexa Fluor-594 goat anti-rabbit (red) (Life Technologies, A-11006 and A-11037) were used as secondary antibodies. Immunostaining was done according to Sagolla et al. [61] on superfrost microscopic slides coated with poly-L-lysine (Sigma, P8920). Preparations were counterstained with DAPI in Vectashield mounting medium (Vector Laboratories, H – 1200) and observed using a IX81 fluorescent microscope (Olympus) equipped with an IX2-UCB camera. Images were processed using Cell^R^ software (Olympus) and ImageJ 1.42q.

## Additional files

Additional file 1: Phylogenetic tree of OTC sequences. The tree based on a 242 positions long protein alignment of 444 sequences was constructed in RAxML using the LG4X + Γ model of substitution. Eukaryotic taxa are highlighted in different colors according to the major group they belong to. The color code is the same as in Fig. 1. The values at nodes represent RAxML bootstrap support/IQ-TREE bootstrap support. Only values above 50 % are shown. Black circles indicate support of 100 %/100 %. Species with multiple sequences included: *Alexandrium tamarense* 1 – CAMPEP 0186340278; *Alexandrium tamarense* 2 – CAMPEP 0186191854; *Alexandrium tamarense* 3 – CAMPEP 0186247540; *Durinskia baltica* 1 – CAMPEP 0200033980; *Durinskia baltica* 2 – CAMPEP 0200081736; *Euglena gracilis* 1 – c20598 g1 i1; *Euglena gracilis* 2 – c34673 g1 i6; *Eutreptiella gymnastica*-like 1 – CAMPEP 0200420840; *Eutreptiella gymnastica*-like 2 – CAMPEP 0200409666; *Karenia brevis* 1 – CAMPEP 0188881430; *Karenia brevis* 2 – CAMPEP 0188950444; *Karlodinium micrum* 1 – CAMPEP 0200795676; *Karlodinium micrum* 2 – CAMPEP 0200767534. The tree is rooted with sequences of bacterial aspartate carbamoyltransferase (ATC; EC 2.1.3.2). (PDF 506 kb)
Additional file 2: Phylogenetic tree of CK sequences. The tree based on a 251 positions long protein alignment of 256 sequences was constructed in RAxML using the LG4X+ Γ model of substitution. Eukaryotic taxa are highlighted in different colors according to the major group they belong to. The color code is the same as in Fig. 1. The values at nodes represent RAxML bootstrap support/IQ-TREE bootstrap support. Only values above 50 % are shown. Black circles indicate support of 100 %/100 %. Vertical black bars indicate well-supported eukaryotic clades: Ch – Chlorophyta; Din – Dinoflagellata; Dip – Diplomonadida; Pa – Parabasalia; Pr – Preaxostyla. Species with multiple sequences included: *Giardia intestinalis* 1 – GSB 16453; *Giardia intestinalis* 2 – GL50803 16453; *Thalassiosira pseudonana* 1 – GI 223995860; *Thalassiosira pseudonana* 2 – GI 224000745; *Trichomonas vaginalis* 1 – TVAG 420500; *Trichomonas vaginalis* 2 – TVAG 261970; *Trichomonas vaginalis* 3 – TVAG 420510. The tree is unrooted. (PDF 482 kb)
Additional file 3: Phylogenetic trees of gene partitions used for concatenation. The values at nodes represent maximum likelihood bootstrap percentages. Eukaryota highlighted in grey. Red taxon names indicate removed sequences. Positions of the particular genes in the alignment: ADI 0–257, OTC 258–499, CK 500–750. The trees are unrooted. (PDF 270 kb)
Additional file 4: Phylogenetic tree of concatenated ADI, OTC, and CK sequences. The tree based on a 750 positions long protein alignment of 67 sequences was constructed in RAxML using LG4X + Γ model. Eukaryotic taxa are highlighted in different colors according to the major group they belong to. The color code is the same as in Fig. 1. The values at nodes represent RAxML bootstrap support/IQ-TREE bootstrap support. Only values above 50 % are shown. Black circles indicate support 100 %/100 %. The tree is unrooted. (PDF 272 kb)
Additional file 5: Phylogenetic tree of concatenated ADI, OTC, and CK sequences with Bacteria and Archaea removed. The tree based on a 750 positions long protein alignment of 15 sequences was constructed in RAxML using LG4X + Γ model. The tree is unrooted. (PDF 2 kb)
Additional file 6: The probability of mitochondrial localization of ADI pathway enzymes in *Monocercomonoides* sp. PA203 and *Paratrimastix pyriformis* as predicted by TargetP and MitoProt II. (DOCX 12 kb)
Additional file 7: Topology of the expected species tree of eukaryotes. The tree is unrooted. (PDF 2 kb)
