# Supplementary figures and images for: Arginine deiminase pathway enzymes: evolutionary history in metamonads and other eukaryotes
Source: BMC Evol Biol. 2016 Oct 6;16:197. doi: 10.1186/s12862-016-0771-4 (PMC5052871; doi:10.1186/s12862-016-0771-4)

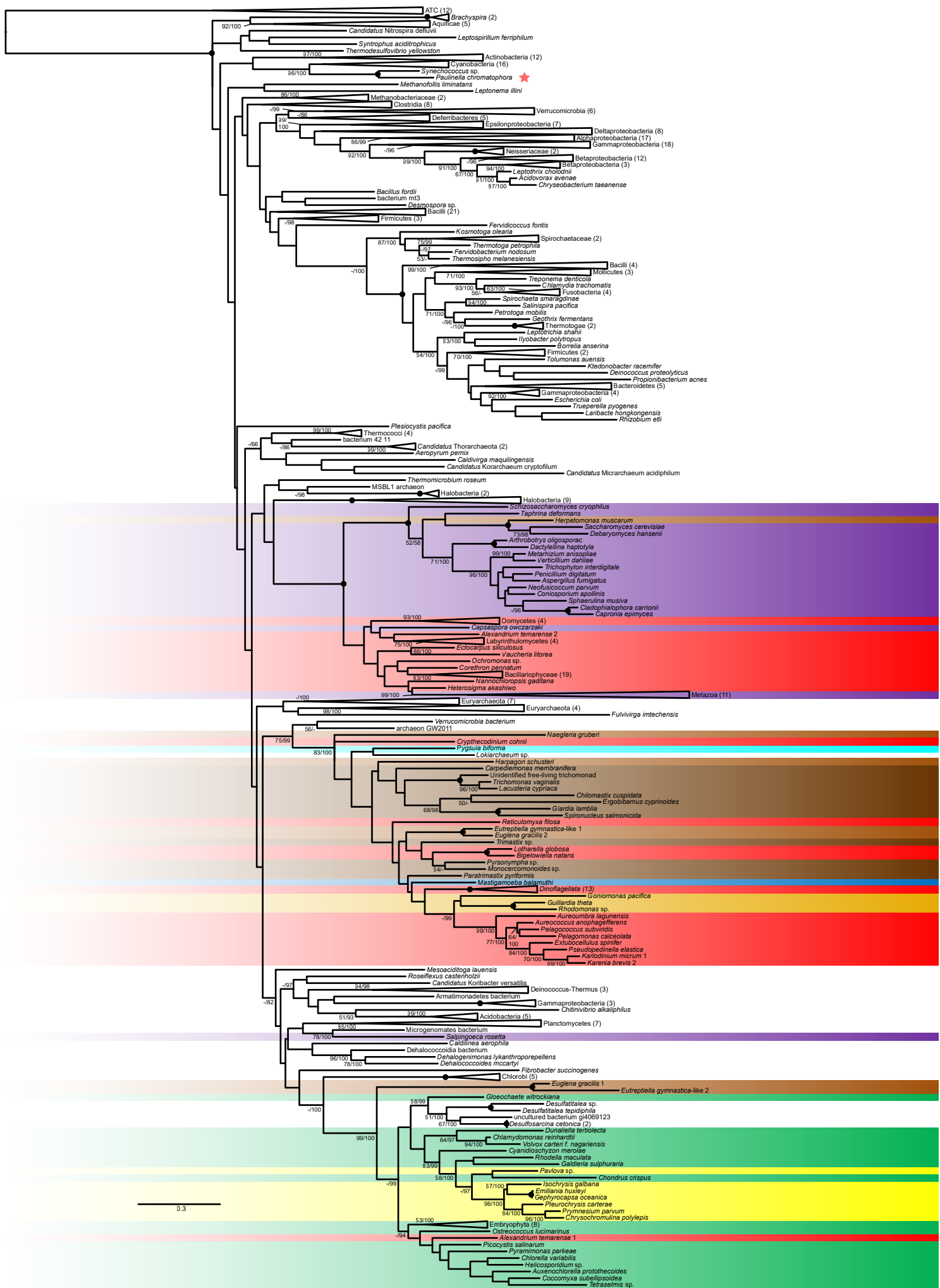

Supplement: Additional file 1: — Phylogenetic tree of OTC sequences. The tree based on a 242 positions long protein alignment of 444 sequences was constructed in RAxML using the LG4X + Γ model of substitution. Eukaryotic taxa are highlighted in different colors according to the major group they belong to. The color code is the same as in Fig. 1. The values at nodes represent RAxML bootstrap support/IQ-TREE bootstrap support. Only values above 50 % are shown. Black circles indicate support of 100 %/100 %. Species with multiple sequences included: Alexandrium tamarense 1 – CAMPEP 0186340278; Alexandrium tamarense 2 – CAMPEP 0186191854; Alexandrium tamarense 3 – CAMPEP 0186247540; Durinskia baltica 1 – CAMPEP 0200033980; Durinskia baltica 2 – CAMPEP 0200081736; Euglena gracilis 1 – c20598 g1 i1; Euglena gracilis 2 – c34673 g1 i6; Eutreptiella gymnastica-like 1 – CAMPEP 0200420840; Eutreptiella gymnastica-like 2 – CAMPEP 0200409666; Karenia brevis 1 – CAMPEP 0188881430; Karenia brevis 2 – CAMPEP 0188950444; Karlodinium micrum 1 – CAMPEP 0200795676; Karlodinium micrum 2 – CAMPEP 0200767534. The tree is rooted with sequences of bacterial aspartate carbamoyltransferase (ATC; EC 2.1.3.2). (PDF 506 kb) [file 12862_2016_771_MOESM1_ESM.pdf]

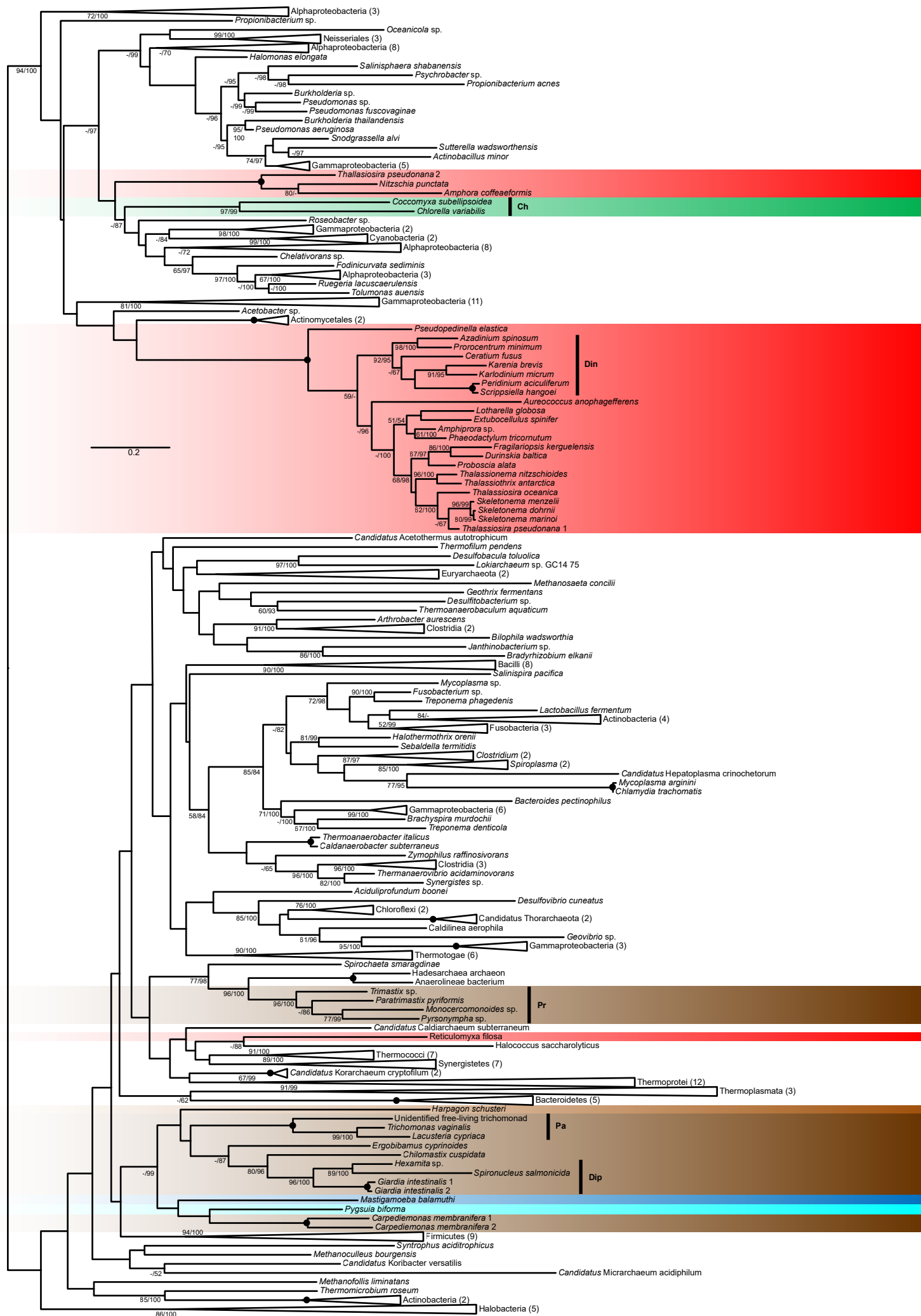

Supplement: Additional file 2: — Phylogenetic tree of CK sequences. The tree based on a 251 positions long protein alignment of 256 sequences was constructed in RAxML using the LG4X+ Γ model of substitution. Eukaryotic taxa are highlighted in different colors according to the major group they belong to. The color code is the same as in Fig. 1. The values at nodes represent RAxML bootstrap support/IQ-TREE bootstrap support. Only values above 50 % are shown. Black circles indicate support of 100 %/100 %. Vertical black bars indicate well-supported eukaryotic clades: Ch – Chlorophyta; Din – Dinoflagellata; Dip – Diplomonadida; Pa – Parabasalia; Pr – Preaxostyla. Species with multiple sequences included: Giardia intestinalis 1 – GSB 16453; Giardia intestinalis 2 – GL50803 16453; Thalassiosira pseudonana 1 – GI 223995860; Thalassiosira pseudonana 2 – GI 224000745; Trichomonas vaginalis 1 – TVAG 420500; Trichomonas vaginalis 2 – TVAG 261970; Trichomonas vaginalis 3 – TVAG 420510. The tree is unrooted. (PDF 482 kb) [file 12862_2016_771_MOESM2_ESM.pdf]

ADI

OTC

CK

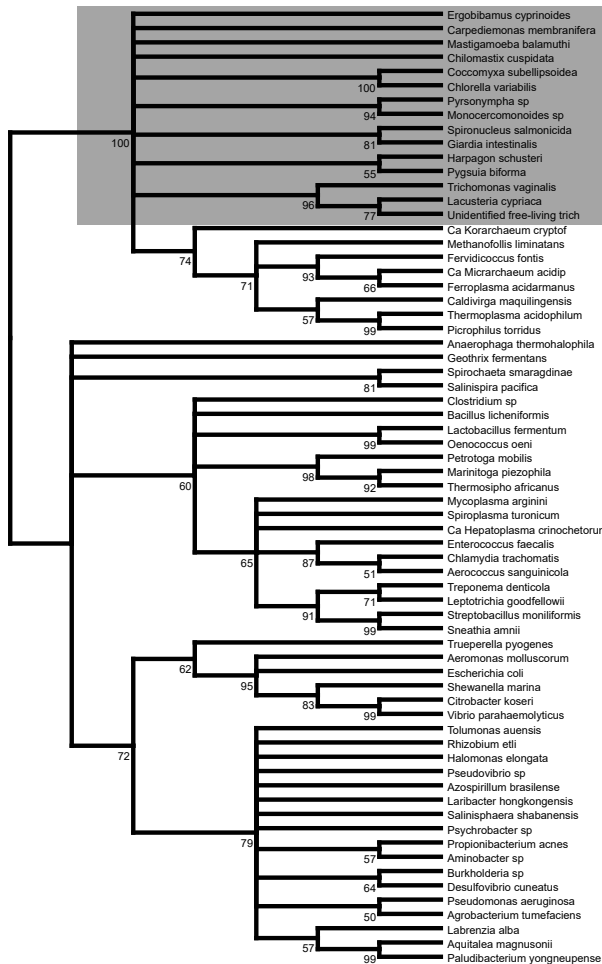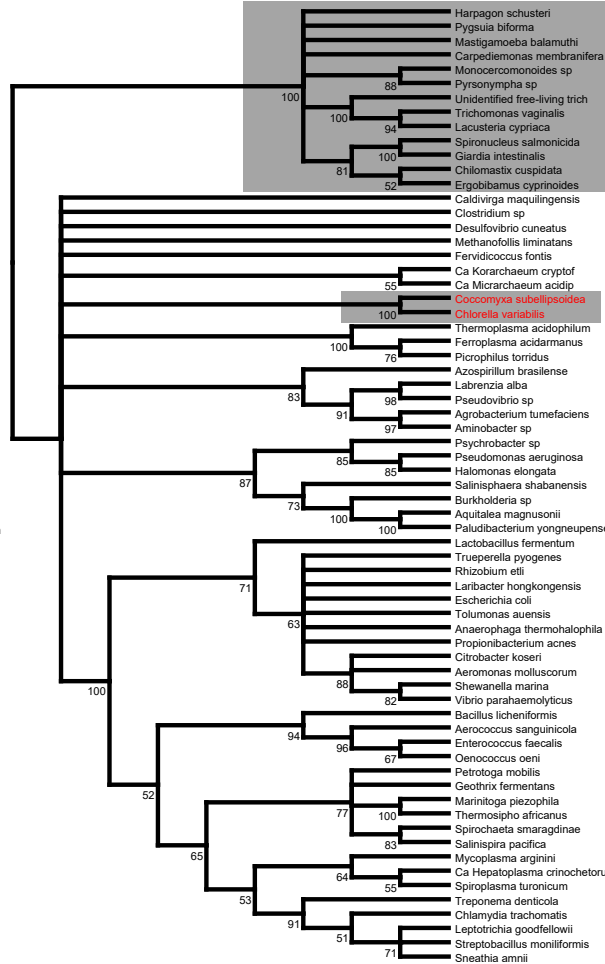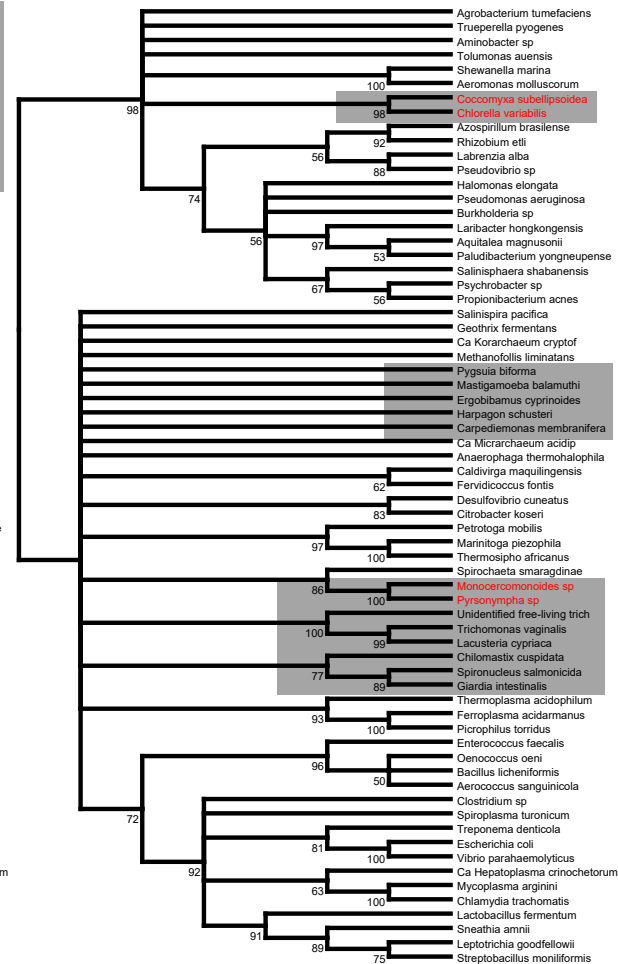

Supplement: Additional file 3: — Phylogenetic trees of gene partitions used for concatenation. The values at nodes represent maximum likelihood bootstrap percentages. Eukaryota highlighted in grey. Red taxon names indicate removed sequences. Positions of the particular genes in the alignment: ADI 0–257, OTC 258–499, CK 500–750. The trees are unrooted. (PDF 270 kb) [file 12862_2016_771_MOESM3_ESM.pdf]

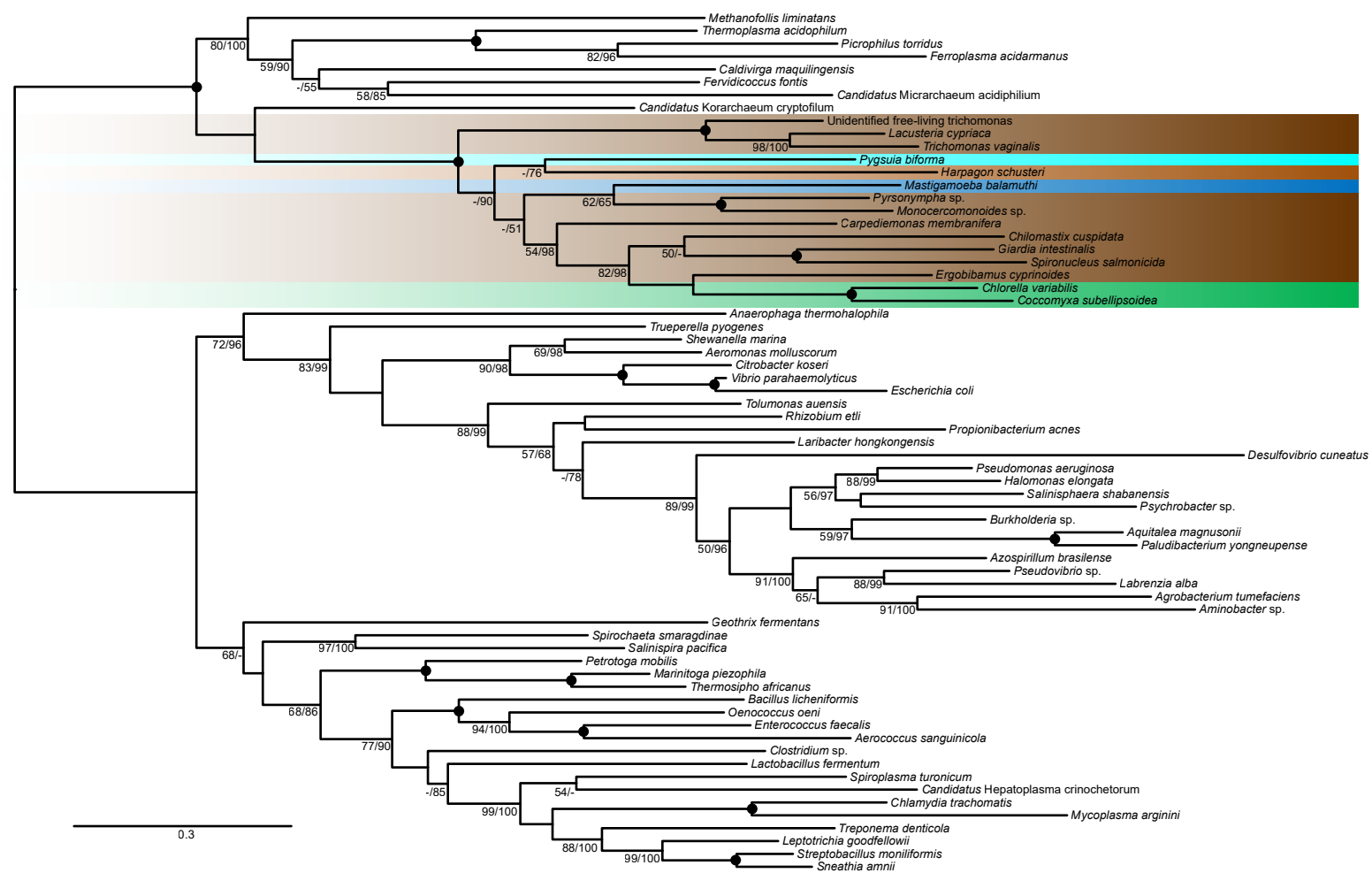

Supplement: Additional file 4: — Phylogenetic tree of concatenated ADI, OTC, and CK sequences. The tree based on a 750 positions long protein alignment of 67 sequences was constructed in RAxML using LG4X + Γ model. Eukaryotic taxa are highlighted in different colors according to the major group they belong to. The color code is the same as in Fig. 1. The values at nodes represent RAxML bootstrap support/IQ-TREE bootstrap support. Only values above 50 % are shown. Black circles indicate support 100 %/100 %. The tree is unrooted. (PDF 272 kb) [file 12862_2016_771_MOESM4_ESM.pdf]

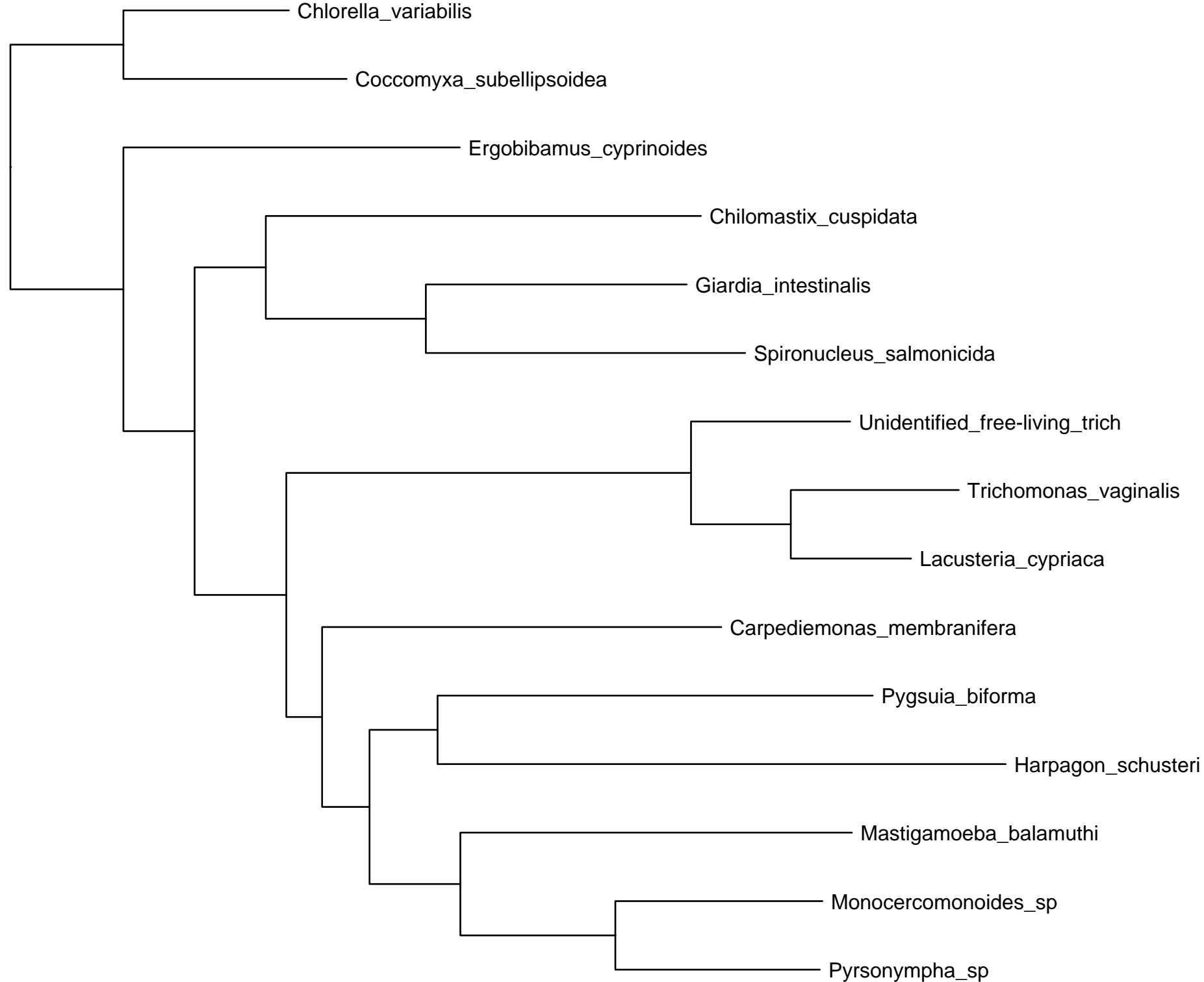

02

Supplement: Additional file 5: — Phylogenetic tree of concatenated ADI, OTC, and CK sequences with Bacteria and Archaea removed. The tree based on a 750 positions long protein alignment of 15 sequences was constructed in RAxML using LG4X + Γ model. The tree is unrooted. (PDF 2 kb) [file 12862_2016_771_MOESM5_ESM.pdf]

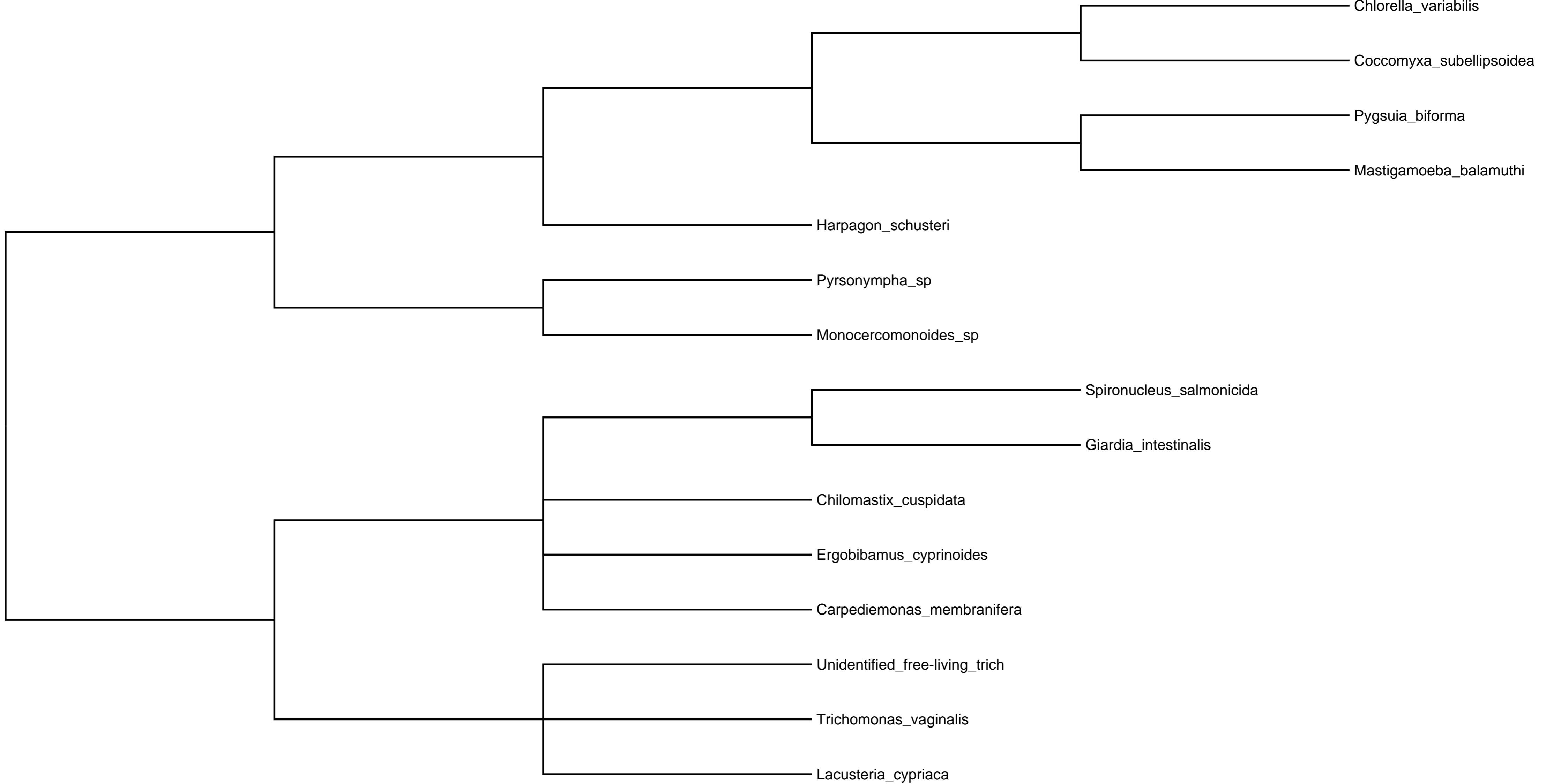

Supplement: Additional file 7: — Topology of the expected species tree of eukaryotes. The tree is unrooted. (PDF 2 kb) [file 12862_2016_771_MOESM7_ESM.pdf]
