# Supplementary material for: Arginine deiminase pathway enzymes: evolutionary history in metamonads and other eukaryotes
Source: BMC Evol Biol. 2016 Oct 6;16:197. doi: 10.1186/s12862-016-0771-4 (PMC5052871; doi:10.1186/s12862-016-0771-4)
Supplement: Additional file 6: — The probability of mitochondrial localization of ADI pathway enzymes in Monocercomonoides sp. PA203 and Paratrimastix pyriformis as predicted by TargetP and MitoProt II. (DOCX 12 kb) [file 12862_2016_771_MOESM6_ESM.docx]

**Additional file 6.** The probability of mitochondrial localization of ADI pathway enzymes in *Monocercomonoides* sp. PA203 and *Paratrimastix pyriformis* as predicted by TargetP and MitoProt II.

| **Species** | **Protein** | **% targetp** | **% mitoprot** | **MTS mitoprot** |
| --- | --- | --- | --- | --- |
| *Monocercomonoides* sp. Pa203 | ADI | 0.055 | 0.0107 |  |
|  | OTC | 0.207 | 0.3934 |  |
|  | CK | 0.161 | 0.22 |  |
| *Paratrimastix pyriformis* | OTC | 0.13 | 0.813 | MPRHLTKISDLS |
|  | CK | 0.069 | 0.0698 |  |
